# Hybrid nanocomposite as a chest wall graft with improved integration by adipose-derived stem cells

**DOI:** 10.1038/s41598-019-47441-9

**Published:** 2019-07-29

**Authors:** Johanna Buschmann, Yoshito Yamada, Konstantin Schulz-Schönhagen, Samuel C. Hess, Wendelin J. Stark, Christine Opelz, Gabriella Meier Bürgisser, Walter Weder, Wolfgang Jungraithmayr

**Affiliations:** 1 Division of Plastic and Hand Surgery, University Hospital Zurich, Zurich, Switzerland; 2 Department of Thoracic Surgery, University Hospital Zurich, Zurich, Switzerland; 3 Institute for Chemical and Bioengineering, Department of Chemistry and Applied Biosciences, ETH Zurich, CH-8093 Zurich, Switzerland; 4 Department of Thoracic Surgery, University Hospital Rostock, Rostock, Germany

**Keywords:** Stem-cell research, Translational research

## Abstract

Surgery of the chest wall is potentially required to cover large defects after  removal of malignant tumours. Usually, inert and non-degradable Gore-Tex serves to replace the missing tissue. However, novel biodegradable materials combined with stem cells are available that stimulate the healing. Based on poly-lactic-co-glycolic acid and amorphous calcium phosphate nanoparticles (PLGA/aCaP) and pure PLGA, a dual layer biodegradable hybrid nanocomposite was generated. Mouse adipose-derived stem cells were cultered on electrospun disks (ASCs of C57BL/6), and biomechanical tests were performed. The cell-seeded scaffolds were engrafted in C57BL/LY5.1 mice to serve as a chest wall substitute. Cell invasion into the bi-layered material, extent of CD45^+^ cells, inflammatory response, neo-vascularization and ECM composition were determined at 1 and 2 months post-surgery, respectively. The bi-layered hybrid nanocomposite was stable after a 2-week *in vitro* culture, in contrast to PLGA/aCaP without a PLGA layer. There was a complete biointegration and good vascularization *in vivo*. The presence of ASCs attracted more CD45^+^ cells (hematopoietic origin) compared to cell-free scaffolds. Inflammatory reaction was similar for both groups (±ASCs) at 8 weeks. A bi-layered hybrid nanocomposite fabricated of electrospun PLGA/aCaP and a reinforcing layer of pristine PLGA is an ideal scaffold for chest wall reconstruction. It is stable and allows a proper host tissue integration. If ASCs are seeded, they attract more CD45^+^ cells, supporting the regeneration process.

## Introduction

Many chest wall discontinuities follow from the removal of aggressive tumors^1,2^, needing adequate covering and chest wall reconstruction^3^. The repaired chest wall should be stable and well integrated into the surrounding tissue. On the one hand, there are different grafts in use: Myocutaneous^4^, muscle^5,6^, pedicled or free myocutaneous or osseomyocutaneous^7^ as well as fasciocutaneous and omental or breast flaps^8,9^. On the other hand, biomaterials offer a viable option – either synthetic^10,11^ or natural like cellulose^12^, among others^13,14^.

Synthetic materials, such as prolene, polypropylene meshes or Gore-Tex, are utilized for chest wall reconstruction in daily clinical practice^15^, also in combination with perforator flaps^16^ or small intestinal submucosa, a natural product that is made cell-free before application^14,17^. Biointegration of chest wall grafts may be improved by cellular therapy, for example by seeding stem cells onto the material. This approach has been delineated for adipose-derived stem cells (ASCs) seeded on artificial hernia^18^, sternal reconstruction^19^ and onto a POC-PLA patch for chest wall repair^20^. Moreover, the application of bone-marrow derived stem cells for sternum reconstruction^21^ as well as osteogenic induced stem cells in chest wall replacement^22^ have been shown to be superior than cell-free approaches.

As a goal to treat full-thickness chest wall defects effectively, the implanted material needs to be firm and surgeon-friendly, fluid- and air-tight as well as biocompatible. During healing, it should degrade without any toxic side products. In addition, fast and full integration into the adjacent tissue should be warranted. At best, the graft evokes cell homing of progenitor cells with a regenerative potential. Biomimetic materials^23^ imitating non-skeletal and skeletal parts of the critical size wound, would enable a rapid and easy involvement of the material into the surrounding tissues. Additionally, adult stem cells might aid the processes of integration, cell homing and/or immunomodulation^24,25^.

Here, we used an adjustable, biodegradable and biocompatible electrospun mesh, composed of two layers, one fabricated from poly(lactic-co-glycolide)/amorphous calcium phosphate (PLGA/aCaP) and the other from pure PLGA, respectively. This hybrid scaffold was intended to be more stable after cell-seeding compared to the mono-layered nanocomposite PLGA/aCaP that has been used as a chest wall graft previously^26^. Our group has shown before that PLGA/aCaP holds great promise in terms of thoracic wall reconstruction^27^. Additionally, the presence of murine ASCs on PLGA/aCaP has been reported earlier to significantly reduce the inflammatory reaction^26^.

ASCs are easily harvested, prevailing in abundance^28^ and adequate proliferation and migration into electrospun meshes composed of PLGA/aCaP has been shown^29,30^. Hence, they represent an ideal stem cell source to seed a chest wall construct.

In order to study the effects of murine C57BL/6 ASCs seeded on a stable, electrospun hybrid material with two layers, to close chest wall defects, PLGA/aCaP scaffolds with ASCs were used as chest wall grafts in a thoracic wall defect C57BL/LY5.1 murine model (endpoints 1 and 2 months). C57BL/LY5.1 mice were used to determine the extent of CD45^+^ cell attraction to the graft. Tensile testing of the novel hybrid scaffold and the two components alone was performed in dry state and after a 2-week incubation in culture medium. Cell densities were assessed (entire cell density, density of endothelial cells, as well as macrophages, lymphocytes and foreign body giant cells); and ECM composition was analyzed by immunohistochemical staining and subsequent histomorphometry.

The hypotheses were that

(i) the cell-seeded hybrid scaffold is more stable than a cell-seeded pure PLGA/aCaP scaffold and thus offers easier handling during implantation,
(ii) ASCs modulate the inflammatory response towards the hybrid scaffold (control cell-free hybrid scaffold),
(iii) ASC-seeded hybrid scaffolds promote the healing process by attraction of CD45^+^ cells and
(iv) ASCs positively influence neo-angiogenesis and ECM remodeling.

## Materials and Methods

### Scaffolds

**Figure 1:**
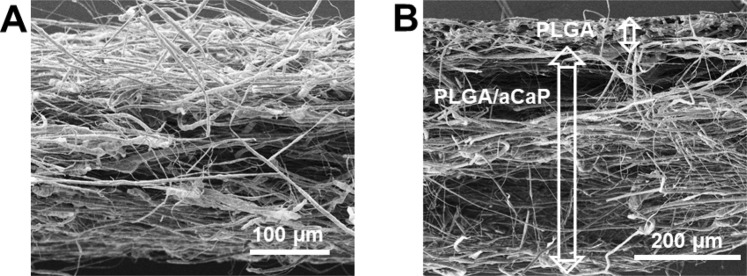
Scaffold characterization. SEM images of the hybrid scaffold at two different magnifications. In (**A**) the fibres of PLGA/aCaP are visible at a 305× magnification; in (**B**) also the thin layer of pure PLGA fibres on top of the PLGA/aCaP fibers is visible (198× magnification).

PLGA (85% lactic acid and 15% glycolic acid, clinically approved) was purchased from Boehringer Ingelheim am Rhein, Germany. The nanoparticles having a Ca:P ratio of 1.5, were received via flame spray pyrolysis as reported by Loher *et al*.^31^. To combine PLGA and the aCaP nanoparticles, the identical protocol was used as reported previously^27^. Hybrid scaffolds were fabricated by electrospinning of two layers; with a 500 µm thick layer of PLGA/aCaP and a 30 µm thick layer of PLGA. The hybrid scaffolds were investigated with scanning electron microscopy (SEM, FEI, Nova NanoSEM 450) (Fig. 1). Transmission electron microscopy (TEM, FEI, Philips CM 12) was used to obtain information regarding particle morphology and to determine the particles’ primary diameter (≈22 nm)^27,29^.

Hybrid scaffold disks with a diameter of 1 cm and and a hight of ≈ 530 µm were prepared. For sterilization, the scaffolds were immersed into a phosphate buffer solution with 200 µg/mL gentamycine (Biowest, Teco Medical, Sissach, Switzerland) and 2.5 µg/mL amphotericine (Biowest, Teco Medical, Sissach, Switzerland) overnight^26,27,29^.

### Adipose-derived stem cells: source and properties

Three specific pathogen-free, male 24 C57BL/6 mice (Harlan, Netherlands) were used to harvest fat tissue samples from abdominal lipectomies^26^. Adipose-derived stem cell extraction was performed according to Zuk *et al*.^32^. Isolated cells were merged and cultured in DMEM medium (Biowest, Teco Medical, Sissach, Switzerland), complemented by 10% of FBS (Biowest, Teco Medical, Sissach, Switzerland) and 50 μg mL^−1^ gentamycine. Passaging of ASCs was performed with 2.5% trypsin/0.23 mM EDTA. Cell were counted with the Neubauer chamber (n = 4)^26^.

Characterization of murine ASCs (extracted from fat of C57BL/6 mice) was based on quantitative RT-PCR; expression levels of CD34, CD44, CD105, ALP, Oct 4, Sox 2, Nanog, Pecam-1, VEGFR1 and VEGFR2 were assessed as described previously^26^.

### Scaffold seeding

The first seeding was realized by carefully placing 10^6^ASCs from P4 to P7 in 100 µL cell culture medium on top of each hybrid scaffold. Subsequently, all seeded scaffolds were cultivated in 6-well plates using 7 mL DMEM medium with 10% of FBS and 50 µg mL^−1^ gentamycine^26^.The second seeding was performed after 24 h, when the scaffolds were turned and seeded with 10^6^ ASCs. Without further turning, the scaffolds were cultivated in a humidified atmosphere of 95% air and 5% CO_2_ at 37 °C for two weeks^26^. Medium was changed every 3 or 4 days. These samples were either fixated overnight by 4% formalin in PBS (Kantonsapotheke Zurich, Switzerland) and then stained (time point 0) or they were used as a chest wall graft *in vivo*. For each condition, there were triplicates (n = 3).

### Animals

Specific pathogen-free, male 24 C57BL/LY5.1) mice (Harlan, Netherlands), weighing 25 g to 29 g, were used. Mice received adequate care in accordance with the Guide for the Use of Laboratory Animals (National Institutes of Health Publication 85-23, revised 1996; Bethesda, MD)^26^. The local animal committee approved the study protocol (Licence of Zürcher Veterinäramt No. ZH002/16).

### Treatment groups

A total of 12 mice were used for the chest wall replacement experiments. Six mice were sacrificed at 4 and six mice at 8 weeks post-operation, respectively. Half of the mice received a cell-free hybrid scaffold, the other half a cell-seeded construct.

### Surgical procedures

All steps of the aforementioned procedure were performed under sterile conditions, using an operating microscope (Wild, Heerbrugg, Switzerland) with 30–40 × magnification^27^. Animals were anesthetized and treated as reported before^27^.

During chest wall reconstruction, animals were placed on a small section of rolled gauze (serving as a counterfort to enhance widening of the intercostal space) on their right side. The lateral left hemi-thorax of the animals was shaved and disinfected^27^. The thoracic wall was freed by ubtle preparation of the subcutaneous and muscle tissue. A piece of 4 mm × 4 mm from the thoracic wall was excised and either a cell-free or a cell-seeded hybrid scaffold was introduced into this gap by running sutures (5-0 prolene, Ethicon, Livingston, Scotland)^27^. No anatomical and physiological change developed because the scaffold was carefully sutured in line with the thoracic wall.

### Histology

After extraction, the graft specimens were fixated in formalin (Kantonsapotheke, Zürich, Switzerland). They were subsequently dehydrated and paraffin-embedded. The sections were 5 µm thick. H&E and Hemalaun-Sudan stainings were performed according to commonly established procedures after deparaffinizing with xylene (Fluka, Switzerland) and rehydrating the sections (descending gradient of ethanol (Fluka, Switzerland)^33^.

### Immunohistochemistry

Immunohistochemistry (IHC) was performed on paraffin sections of formalin fixated tissues, with a Ventana Benchmark automated staining system (Ventana Medical Systems, Tucson, Arizona). For antigen retrieval, slides were heated with cell conditioner 1 (standard procedure). Endogenous biotin was blocked with the appropriate kit^26^. Primary monoclonal rat antibody against F4/80 (BMA Biomedicals AG, clone BM8, dilution 1:200), rabbit antibody against CD3 (DAKO, clone SP7, dilution 1:300), CD31 (Abcam Limited, polyclonal, dilution 1:50), α-smooth muscle actin (DAKO, dilution 1:2), collagen I (Abcam Limited, dilution 1:100), CD45 (BD Pharmingen, dilution 1:50) and fibronectin (Millipore, dilution 1:200) were applied and revealed with the iVIEW DAB detection kit, yielding a brown reaction product. The signal was enhanced with the Ventana amplification kit. Prior to glass coverslipping, the slides were counterstained with hematoxylin (HT)^26^.

Immunohistological analysis was performed with ImageJ 1.51t16. Details for the different IHC stainings: alpha-SMA, collagen I, fibronectin and F4/80 relative intensities were assessed by defining the scaffold area in the tissue sections. Subsequently the pixles stained with Hematoxylin and DAB were detected using QuPath v 0.1.2. The percentage of DAB stained pixels of the total stained area (Hematoxylin & DAB) were plotted. Always two tissue section per mice were averaged. Vascularization was counted in five zones. The images were color de-convoluted and converted to a binary image. The vessels were detected with the particle count function of ImageJ. Lymphocytes were counted in five zones. The lymphocytes were detected with the particle count function of ImageJ. CD45 intensity was scored in 60 fields of view (FOV) for semi-quantitative assessment, while CD45+ cell density was determined in fifteen FOV.

### Cell counting in different local zones

Six zones (z1–z6) were defined starting from the outer periphery: z1 = 0–25 µm, z2 = 25–50 µm, z3 = 50–75 µm, z4 = 75–100 µm, z5 = 100–150 µm, z6 = 150–350 µm with fifteen FOV for each zone, time point and side (towards skin and lung)^27^.

### Tensile testing

Bulk mechanical properties of the pure PLGA, pure PLGA/aCaP and the hybrid meshes were determined (n = 5). Stress-strain curves of the scaffolds were recorded and ultimate stress (failure stress [MPa]) was evaluated. The tests were performed at time point 0 in dry state and after 2 weeks of incubation in DMEM culture medium in wet state.

### Statistical analysis

All data were analyzed with StatView 5.0.1 software. One-way statistical analysis of variance (ANOVA) was conducted to test the significance of differences between different groups, time points and local zones. Pairwise comparison probabilities (*p*) were calculated using the Fisher’s PLSD post hoc test to test for differences between the groups^27^. *P* values < 0.05 were considered significant and marked by (*), p < 0.01 (**) and p < 0.001 (***). Values were expressed as means ± standard deviations.

## Results

### Tensile strength of hybrid scaffold

**Figure 2:**
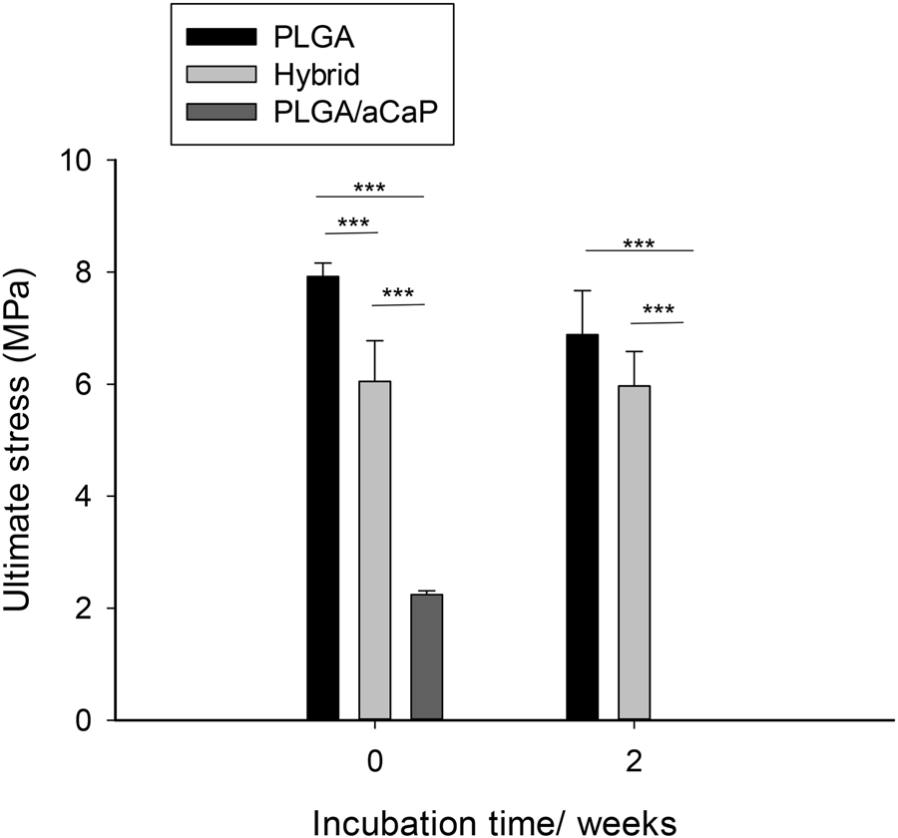
Mechanical properties. Ultimate tensile stress of dry electrospun (incubation time = 0) and of wet scaffolds after a 2-week incubation in DMEM culture medium.

One of the main reasons to further develop our chest wall graft material used previously cell-free^27^ and ASC-seeded^26^ was the fact that two weeks of cell seeding and cultivation *in vitro* prior to implantation changed the mechanical stability drastically resulting in weak cell-seeded grafts. Hence, pure PLGA, pure PLGA/aCaP and newly developed hybrid meshes (PLGA-aCaP/PLGA) were used for tensile testing. While pure PLGA had the highest failure stress, followed by the hybrid mesh, both being significantly higher than pure PLGA/aCaP mesh, a 2-week incubation in DMEM lowered the maximum strength for all three scaffold materials; in case of PLGA and hybrid scaffold only to a minor extent. For PLGA/aCaP, however, the ultimate strength was significantly reduced to zero (Fig. 2).

### Scaffold integration

**Figure 3:**
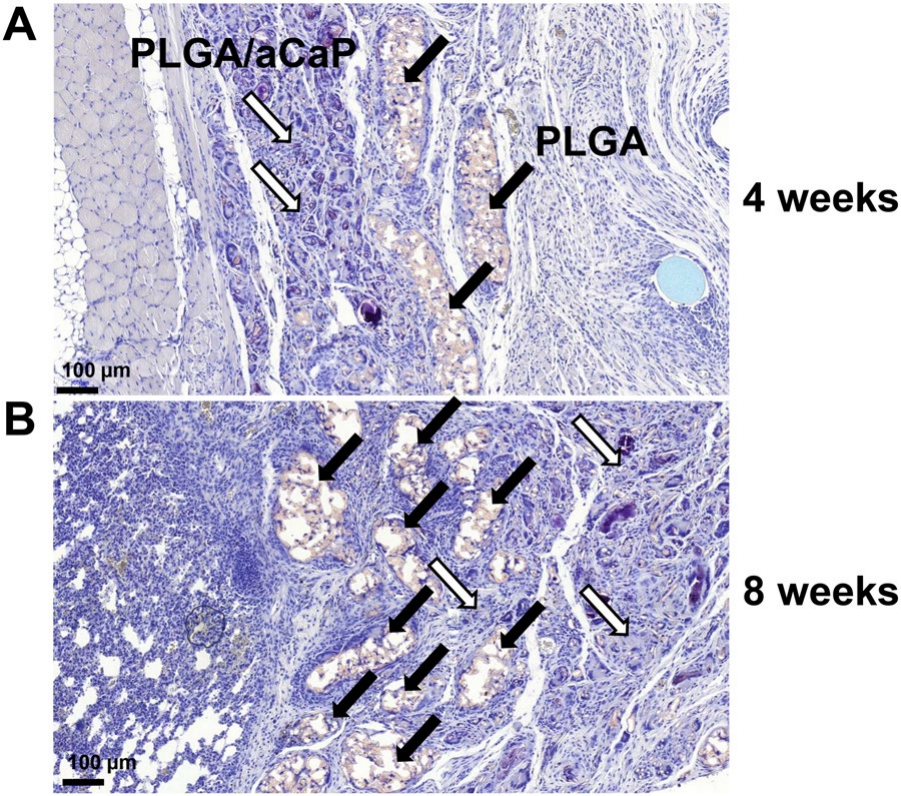
Scaffold degradation *in vivo*. Hemalaun Sudan stained section of ASC-seeded scaffolds 4 and 8 weeks post-operation, respectively. Black arrows depict degrading PLGA areas and white arrows PLGA/aCaP. Note, PLGA builds up discrete pieces (larger at 4 weeks, smaller at 8 weeks), while PLGA/aCaP is completely and homogenously invaded by cells.

The hybrid scaffold was very well integrated into the surrounding tissue, for both, cell-seeded and cell-free scaffolds and from the skin as well as from the lung side, respectively. The thin reinforcing PLGA layer had started to degrade at 4 weeks and even more so at 8 weeks post-operation, leaving behind a layer broken up into discrete pieces of varying size (Fig. 3). As for the PLGA/aCaP thicker layer of the hybrid scaffold, cells had completely invaded all pores between the electrospun fibers.

### Total cell density

**Figure 4:**
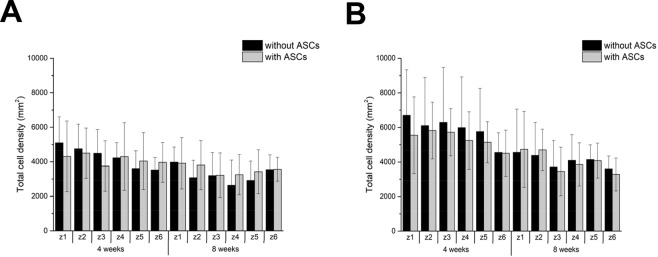
Cell distribution. Entire cell density as a function of period post-surgery and local sector for cell-free hybrid scaffolds and for ASC-seeded grafts, respectively, and from the skin side (**A**) and the lung side (**B**). No statistically significant differences in total cell density were found between scaffolds with and without ASCs.

The number of all cells per area is given for different periods post-operation and local sectors in the scaffolds with ASCs compared to scaffolds without ASCs (Fig. 4). The number of all cells per area at 8 weeks post-surgery was similar for scaffolds with and without ASCs, respectively, and they were homogenously distributed over all layers.

### Cells of hematopoietic origin: CD45^+^

**Figure 5:**
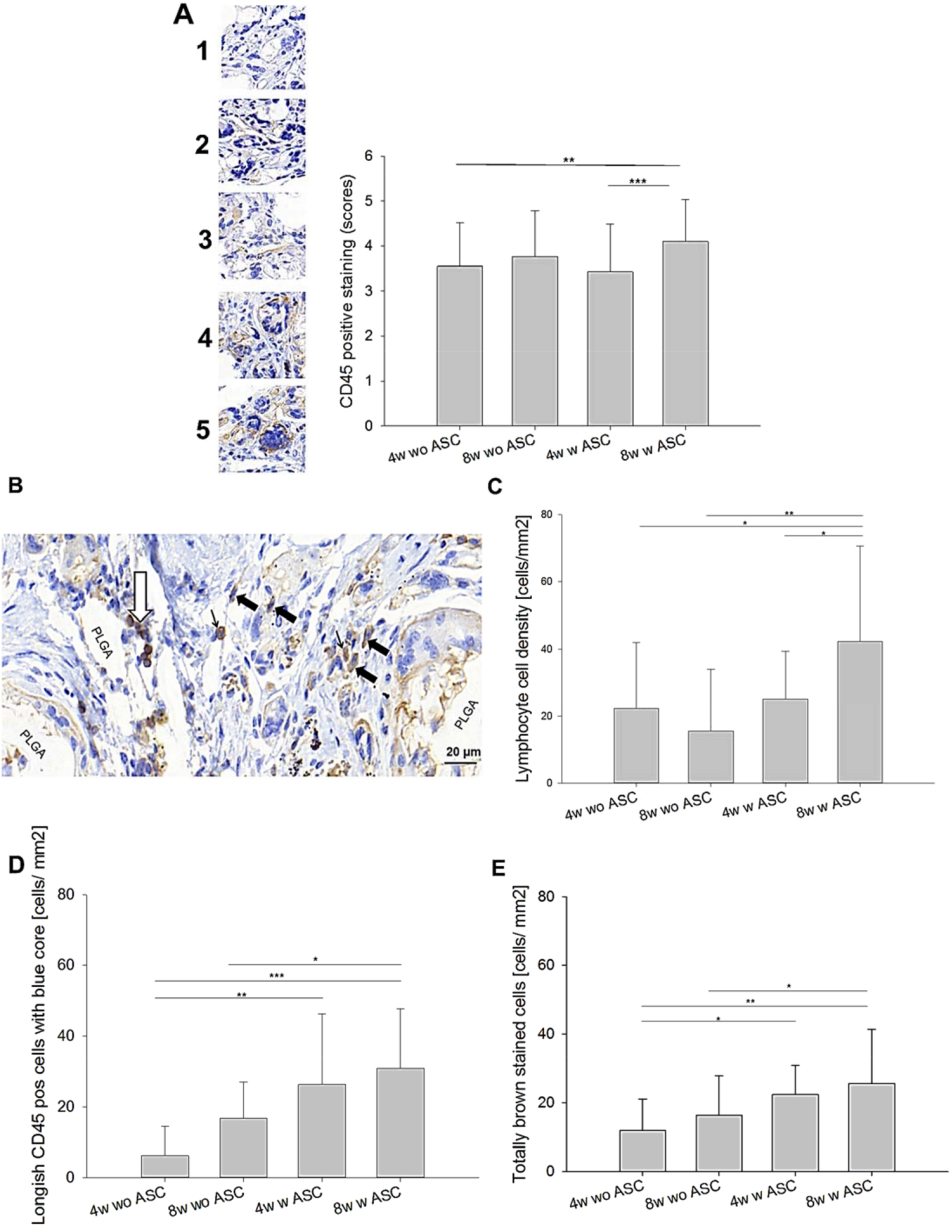
Cells of hematopoietic origin. Semi-quantitative analysis of CD45 positive staining. Representative examples for each score and data for *in vivo* outcome (top, **A**). Histological section to exemplify cell types with different brown intensities within the cells (middle left, **B**); Key: white arrow = CD45^+^ lymphocytes; bold black arrow = spindle-shaped cells CD45^+^ cells with blue core; and thin black arrow = totally brown stained CD45^+^ cells. Analysis of CD45^+^ lymphocytes (middle right, **C**), spindle-shaped cells CD45^+^ cells with blue core (lower left,D) and spindle-shaped CD45^+^ cells without blue core (lower right, **E**). *Key*: wo = without; w = with; 4w = four weeks; 8w = 8 weeks.

CD45 staining revealed an increase in CD45^+^ cells at 8 weeks compared to 4 weeks for the ASC-seeded scaffolds, while it was not significantly increased in the hybrid scaffold without ASCs (Fig. 5). Moreover, the semi-quantitatively determined CD45 intensity was significantly higher at 8 weeks for cell-seeded materials as compared to scaffolds without cells at 4 weeks. Three cell types, CD45^+^ stained, were analyzed quantitatively: (i) roundish lymphocytes with a thin brown membrane and a blue core were found to be significantly increased at 8 weeks in the ASC-seeded samples, confirming findings by a more detailed, zone- and side-specific analysis of lymphocytes by CD3 staining (see below); (ii) spindle-shaped cells CD45 cells with a blue core and (iii) spindle-shaped CD45^+^ cells without blue core.

### Fibrosis

**Figure 6:**
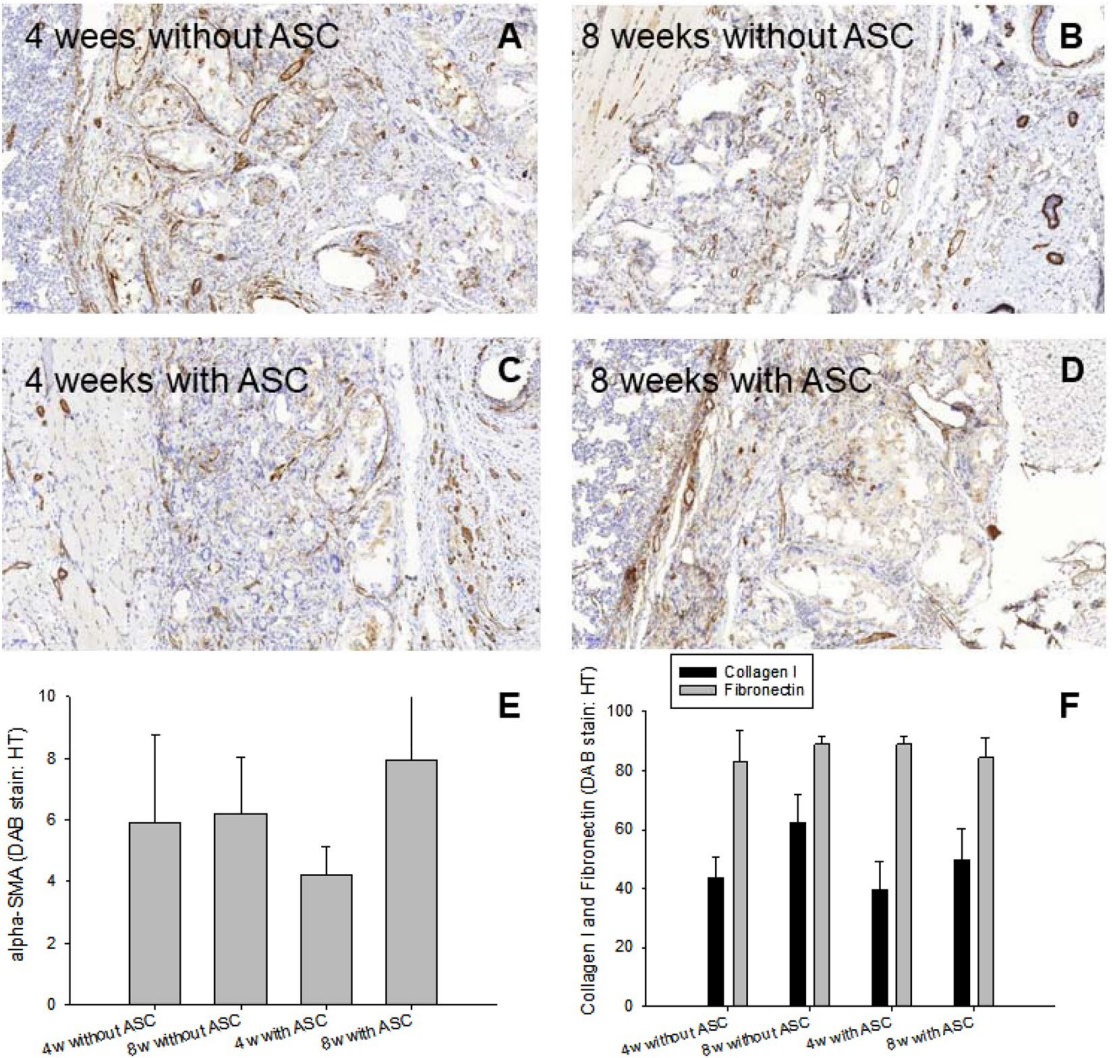
Fibrosis. α-SMA staining in representative histological sections at two time points (**A**–**D**), semi-quantitative analysis of α-SMA intensity (**E**) and collagen I and fibronectin intensities in immunohistochemically stained sections (**F**). *Key*: 4w = four weeks; 8w = 8 weeks, DAB = DAB staining, HT = hematoxylin.

α-SMA staining revealed no statistically significant differences, neither between the ASC-seeded and the cell-free scaffolds nor as a function of time (Fig. 6).

### Extracellular matrix

Extracellular matrix composition was analyzed by collagen I und fibronectin staining (Fig. 6). For collagen I, a significant increase between 1 and 2 months was found for the cell-free scaffolds, but not for the ASC-seeded ones. As for fibronectin, no significant differences were observed; neither for the impact of cell seeding nor time point post-operation.

### Inflammatory reaction

The fraction of F4/80^+^ macrophages of the total cell number is shown in SI Fig. 1. Although there was no significant increase over time, for both, the cell-free and the cell-seeded scaffolds, respectively, scaffolds with ASCs exhibited higher percentages of macrophages compared to cell-free ones.

**Figure 7:**
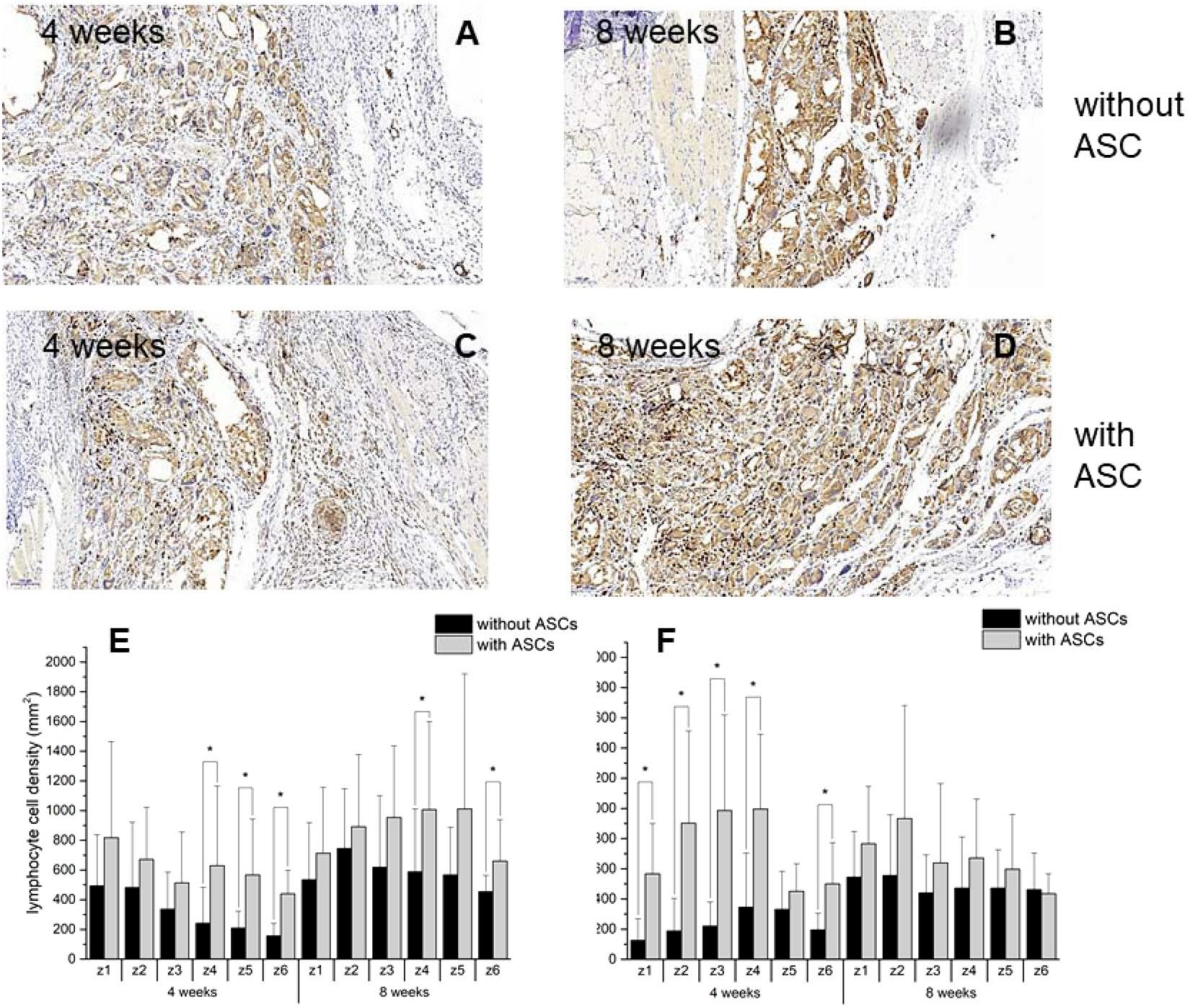
Lymphocytes. Typical CD3 stained histological sections and densities of lymphocytes at two different time points (**A**–**D**) stained positive for CD3 in different zones from skin side (**E**) and from lung side (**F**).

CD3^+^ lymphocytes were present under both conditions; in chest wall grafts with and without cells (Fig. 7). While ASC-seeded scaffolds were infiltrated by significantly more lymphocytes at 4 weeks and for both sides (from the skin side and the lung side, respectively), this effect completely vanished at 8 weeks for the lung side, and was only observed for two distinct zones inside the material on the skin side.

### Neo-angiogenesis

**Figure 8:**
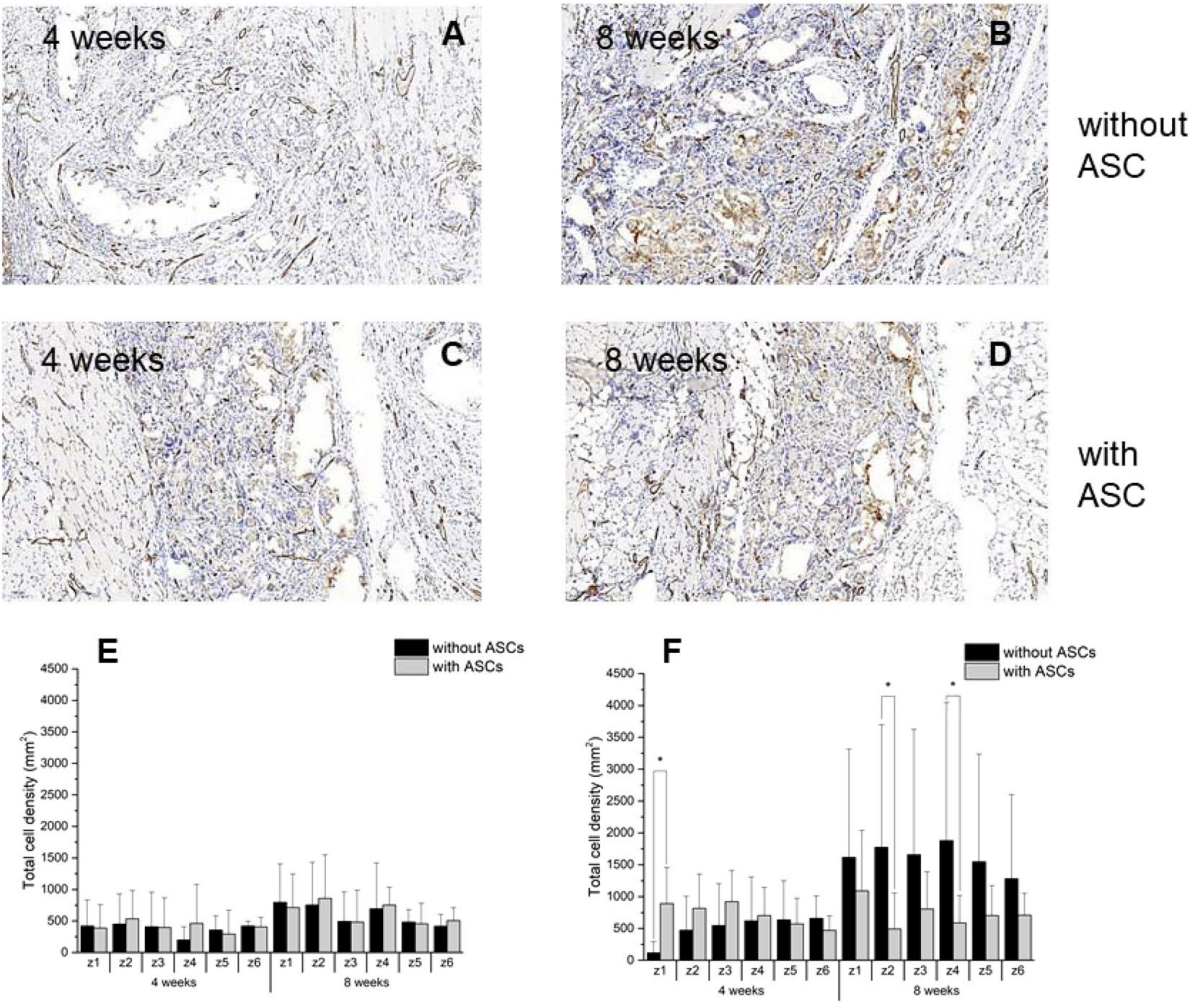
Endothelial cells. Typical CD31 stained histological sections and densities of endothelial cells at two time points (**A**–**D**) stained positive for CD31 in different zones from skin side (**E**) and from lung side (**F**).

In Fig. 8, the number of CD31^+^ stained cells were assessed for the defined zones on the skin as well as on the lung side for both time points and ±ASCs. While there were no significant differences on the skin side, the analysis of the lung side revealed significantly moreCD31^+^ cells for the grafts without cells at 8 weeks. Only in z1 and at 4 weeks, it was the opposite – with significantly more CD31^+^ cells for ASC-seeded grafts.

## Discussion

Previously, our research team has demonstrated that cells infiltrate better into a porous electrospun PLGA/aCaP nanocomposite (without seeded cells) used as a chest wall graft in mice compared to the non-porous inert Gore-Tex material that is currently used as a gold standard^27^. In addition, the new vasculature was uniform throughout the PLGA/aCaP material, while almost no vessels infiltrated into Gore-Tex^27^. Therefore, electrospun PLGA/aCaP nanocomposite was considered a convenient novel scaffold for thoracic wall reconstruction.

In a subsequent study, PLGA/aCaP was seeded with ASCs, leading to a reduced inflammatory reaction towards the material as assessed by F4/80^+^ macrophages and CD3^+^ lymphocytes^26^. The major drawback, however, was the insufficient stability of PLGA/aCaP after cell seeding and a 2-week *in vitro* cultivation, which was resolved at that time by adding a cell-free nanocomposite layer that had not experienced contact with cell culture medium before^26^.

To overcome the problem of graft instability, we developed a novel, more stable hybrid scaffold for chest wall repair, consisting of electrospun PLGA/aCaP and pure PLGA (Fig. 1), the latter realized as a thin reinforcing layer. Upon cell seeding, this hybrid scaffold beholds its initial stability (Fig. 2) and is easily handled during implantation, especially during suturing.

In contrast to ASC-seeded pure PLGA/aCaP nanocomposite^26^, however, we found a significantly increased CD3^+^ lymphocyte cell density 4 weeks post-operation (Fig. 8). Such increased lymphocyte accumulation stand in accordance with Gong and co-workers, who determined more CD3^+^ cells in human ASC-treated group compared to control (cell-free)^34^. We speculate that PLGA releases hydrogen cations resulting in a local pH drop^35^, which might be affecting the sensitive ASCs, resulting in an *in situ* lymphocyte accumulation. This effect vanished at 8 weeks post-operation, confirming an only transiently increased inflammatory reaction in the ASC-seeded hybrid scaffolds.

In accordance to our previous findings^26^, the ASC-seeded hybrid nanocomposite showed lower numbers of CD31^+^ cells than the cell-free scaffold, implying fewer and/or smaller vessels in the cell-seeded graft (Fig. 8): ASC seeding increases cell densities in the pores of the electrospun scaffolds, which might hinder new vessel ingrowth by steric hindrance. Nevertheless, compared to totally avascular Gore-Tex^27^, both, the cell-free and the ASC-seeded novel hybrid scaffolds were well and homogenously vascularized.

The patient group that profits most from that kind of chest wall reconstruction is that with a malignant chest wall tumour, either arising from the chest wall itself or from a lung cancer that is infiltrating the chest wall. But how to choose an appropriate material to close such chest wall defects? The heterogeneous structure of the chest wall, including soft tissue, cartilage and bone, demands for materials allowing the reconstruction of tissues with a wide range of stiffness, topography and composition. A viable option is to use vascularized composite allografts^36^, but also the application of cell-free grafts derived from different tissues is suitable. Due to its integrative and flexible properties, dermis had been successfully applied to close thoracic wall defects. Among others, *Permacol*, a decellularized porcine dermal collagen matrix properly covered thoracic wall defects in children undergoing resection of malignancies^37^ - without any adverse reactions. In addition, a good material for thoracic wall replacement should be stable and needs to seal the pleura, thus should be well covered with connective tissue. Additionally, it should be flexible enough to be properly fixed at the edges of the defect. Finally, it should protect the viscera and prevent infection^15^.

Biological grafts serve these claims, such as muscle^6^, myocutaneous flaps or fascias^38–41^. In addition, traditional hernia repair pieces have proven to successfully close the chest wall after tumour resection (chondrosarcoma) as reported in a case study^42^. In order to strengthen the hernia, a synthetic Dacron patch (made of polyethyleneterephtalate (PET) fibres) was fixed on top, exemplifying that biological and synthetic materials go hand in hand in the operation room^42^.

Other synthetic polymers like poly-lactic-acid (PLA) are appropriate materials^15^, and metal plates of titanium coated with acellular collagen matrix have rebuilt the thoracic wall integrity with a satisfactory outcome^43^. Chest repair by means of prosthetic metal materials, such as steel or titanium, is also used: Matrix Rib titanium plates were successfully applied in patients suffering from sternocostal non-union, pseudoarthrosis or dislocated ribs^44^. Recently, 3D printing approaches have been investigated and may deliver next generations materials for chest wall reconstruction^45–47^. Reviews that present suitable graft materials, the choice of which may be driven by cost effectiveness^48^, and the surgeon’s preference^49,50^. There is no consensus which graft material is the best one.

Electrospun PLGA/aCaP was chosen as this material is fabricated from a ceramic (amorphous calcium phosphate nanoparticles) and an organic phase (PLGA) – enabling bio-mimesis of different tissues that are part of the thoracic wall; bone, cartilage and connective tissue. The inorganic nanoparticles are transformed into hydroxyapatite (HAp), which in turn stabilizes the graft. An important characteristic of (the *in situ* generated) HAp is its tight binding to collagen in the soft tissue, based on covalent bonds between hydroxyl groups and stromal collagen. Furthermore, in contact with bone, HAp binds to collagen fibrils, forming a direct bond to bone^51–53^. Thus, this bioactive nanocomposite allows both, a stable integration in soft tissue as well as in hard tissue like sternum.

The highly porous structure of PLGA/aCaP enables also an easy vascularization^27^. Successful previous studies have reported that PLGA/aCaP had been applied as a bone void filler during calvarial repair in a rabbit model showing sufficient and homogenous vascularization^54^ and in long bone critical size defects in sheep^55^.

Why might ASCs improve the nanocomposites functionality? As multipotent cells, ASCs can differentiate towards various cell types, such as bone cells, cells in cartilage and vessels^28^. They are therefore offering versatile functions at different places of the chest wall graft. Furthermore, ASCs regulate the immune system^56,57^, and their anti-inflammatory action^58^ can be of advantage.

Here, ASCs attracted more cells of hematopoietic origin to the chest wall defect compared to cell-free grafts: there were more CD45^+^ lymphocytes, but also spindle-shaped CD45^+^ cells. This implies a better remodelling via blood-supplied cells compared to cell-free grafts. It is especially noteworthy to mention, because ASC-seeded grafts exhibited lower CD31^+^ cell densities, in other words, less vessels that allowed the recruitment of CD45^+^ cells to the repair site. We were not able to further identify the CD45^+^ spindle-shaped cells, which had either a blue core (not CD45^+^) or a throughout brown colour (all CD45^+^). It might be speculated that these cells with an obviously a hematopoietic origin (from Ly5.1 CD45^+^ mice), were de-differentiated monocytes or epithelial precursor cells. The ASC-seeded grafts must have attracted these kind of cells to the repair site by paracrine function, because ASCs do not remain for 8 weeks at the site of action, but are usually washed out earlier. It was reported that ASC-seeded polysiloxane/collagen scaffolds did not have any ASCs left on the scaffold after a 30-day period^59^.

Therapeutic approaches based on ASCs can affect lung inflammation. COPD, the chronic obstructive pulmonary disease, was induced in a pig model with smoke from cigarettes. ASC therapy decreased tracheal hyper-responsiveness and lung inflammation^58^. The paracrine effect of ASCs observed as suppressed allergic airway inflammation has been attributed to PGE_2_ (prostaglandin E2) and TGF-β (transforming growth factor beta), both secreted by ASCs^60^. Furthermore, ASCs not only decreased inflammatory response in white adipose tissue of a mouse model, but also led to a switch of M1 to M2 macrophages by paracrine function^61,62^. It is therefore not surprising that ASCs that were applied in an inflammation model of rats suppressed pro- and enhanced anti-inflammatory cytokines^63^.

Although all these examples show a positive effect in terms of restricting inflammatory response, we found a slightly higher inflammatory reaction towards our novel hybrid material, as macrophages were increased in the ASC-treated graft at 8 weeks (Fig. 7). It has to be emphasized, however, that F4/80 staining to assess macrophages covers both, M1 and M2-like subtypes. Therefore, the increased macrophages in the ASC-treated graft at 8 weeks may include more M2 than M1 phenotypes. Although not significantly different from ASC-seeded scaffolds at 4 weeks, this trend towards higher inflammatory cell densities was also reflected in lymphocytes. As we had found lower inflammatory reaction for the pure PLGA/aCaP mesh used as chest wall graft and seeded with ASCs in a previous work^26^, the small increase in the inflammatory reaction found here could be attributed to the reinforcement of the chest wall graft by the pristine PLGA mesh – which is known to evoke local pH drops that in turn stimulate an inflammatory response.

There are many reports that show the advantageous impact on angiogenesis and vascularization by ASC therapy or ASC-seeded delivery devices and biomaterials^64–67^.

As for the hybrid nanocomposite used here, the grafts with stem cells had smaller endothelial cell densities compared to PLGA/aCaP without cells (Fig. 8). This stands in accordance with previous findings in our group^26^ and can be explained by steric hindrance: The cellular density in grafts with cells was so high that neo-vascularization was more challenging compared to scaffolds without cells, exhibiting open pores that were easily to access and infiltrate. Still, compared to the gold standard Gore-Tex, our novel ASC-seeded hybrid scaffold allowed significantly more vessel ingrowth because Gore-Tex does not allow any vessel to grow into the inert material.

A hybrid scaffold made of electrospun PLGA/aCaP and a layer of pristine PLGA is a superior biomaterial for chest wall reconstruction compared to PLGA/aCaP without PLGA. The main and important advantage is its preserved mechanical stability, even after a two-week cell culture *in vitro*, allowing an easy application of ASCs or other cells in combination with the hybrid material. *In vivo*, this novel hybrid material successfully closes and repairs critical size chest wall defects in mice. The seeding of ASCs onto the hybrid scaffolds leads to an enhanced recruitment of hematopoietic cells, supporting the regeneration at the defect site. The technically easy isolation of ASCs from fat allows a viable option to seed such scaffolds and use them to close and reconstruct chest wall defects, e.g. after tumour resection in clinics which at the same time is the main indication for thoracic wall resection. Hence, patients with aggressive tumours would be the group of patients that would benefit from such a treatment. Although mild increased inflammatory response, ASC-seeded hybrid scaffold offers everything a thoracic surgeon needs to close a chest wall defect – a stable, easy to handle material, which can be adjusted flexibly to the shape of the defect, is adequately vascularized and biodegradable over time, paving its way for future clinical application.

## Supplementary information

Supplementary Information
