# Supplementary material for: Hybrid nanocomposite as a chest wall graft with improved integration by adipose-derived stem cells
Source: Sci Rep. 2019 Jul 29;9:10910. doi: 10.1038/s41598-019-47441-9 (PMC6662805; doi:10.1038/s41598-019-47441-9)
Supplement: Supplementary file 1 — Supplementary Information [file 41598_2019_47441_MOESM1_ESM.pdf]

Supporting Information

# Hybrid nanocomposite as a chest wall graft with improved integration by adipose-derived stem cells

Johanna Buschmann, Ph.D.*¤[1], Yoshito Yamada, M.D.¤[2], Konstantin Schulz-Schönhagen¤[3], Samuel C. Hess, Ph.D.[3], Wendelin J. Stark, Ph.D.[3], Christine Opelz[2], Gabriella Meier Bürgisser[1], Walter Weder, M.D.[2], Wolfgang Jungraithmayr, M.D., Ph.D. [2,4]

¤ These authors contributed equally to the study.

[1]Division of Plastic and Hand Surgery, University Hospital Zurich, Switzerland

[2]Division of Thoracic Surgery, University Hospital Zurich, Switzerland

[3]Institute for Chemical and Bioengineering, Department of Chemistry and Applied Biosciences, ETH Zurich, CH-8093 Zurich, Switzerland

[4]Department of Thoracic Surgery, University Hospital Rostock, Rostock, Germany

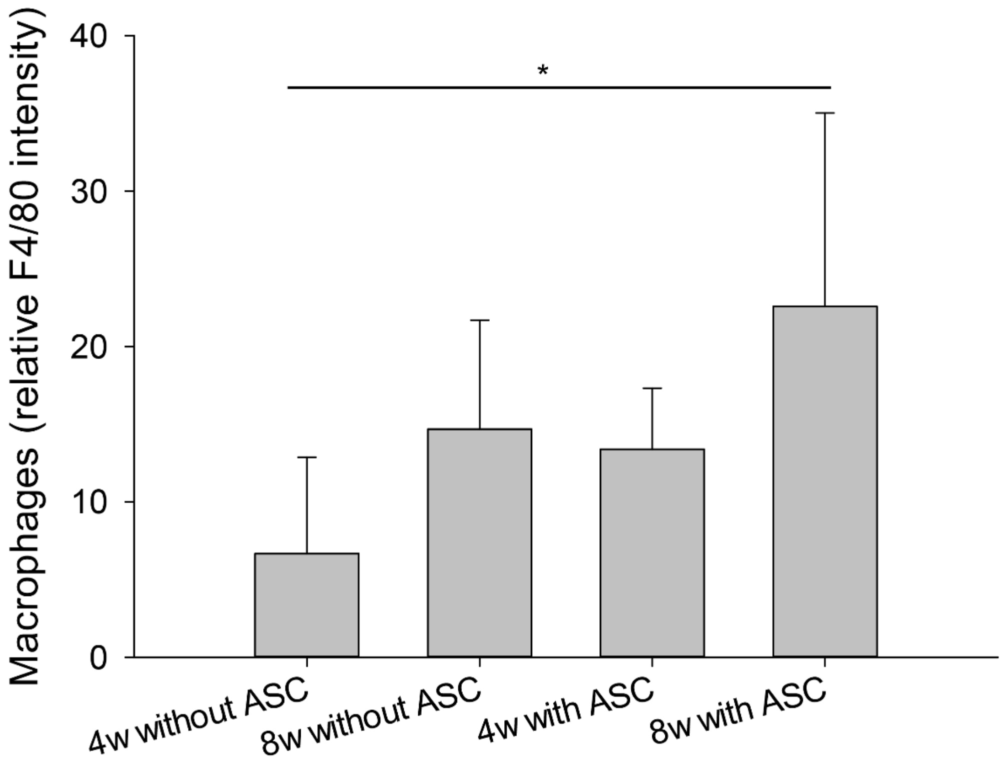


**Figure S1**: Inflammatory response: macrophages. Percentage of **F4/80+ macrophages** as compared to total cell densities. *Key*: 4w = 4 weeks; 8w = 8 weeks.
